# Optimizing nursing care for neutropenic patients: a holistic perspective on education, support, and clinical management

**DOI:** 10.3389/fmed.2025.1633708

**Published:** 2025-10-13

**Authors:** Zhao-ning Xu, Li-ping Gao, Xin-kai Wang, Xiu-li Yu, Yue Cong, Peng-ju Wang

**Affiliations:** ^1^Department of Nursing Care, The First Hospital of Jilin University, Changchun, China; ^2^Department of Central Sterile Supply, The First Hospital of Jilin University, Changchun, China; ^3^Department of Intensive Care Unit, The First Hospital of Jilin University, Changchun, China; ^4^Department of Gynecology, The First Hospital of Jilin University, Changchun, China

**Keywords:** neutropenia, nursing care, education, clinical management, mini-review

## Abstract

Neutropenia, characterized by neutrophil counts below 1,500 cells/μL, increases infection risk and worsens treatment outcomes in oncology and immunocompromised patients. This paper outlines evidence-based nursing strategies in four domains: (1) Clinical management with strict infection control, risk tools, and targeted drug therapies; (2) Patient education through competency-based training and mHealth tools, improving adherence by 42%; (3) Psychosocial support using telehealth and model of Managing Cancer And Living Meaningfully therapy, reducing distress by 38–45%; and (4) Interdisciplinary care with structured pathways lowering complications by 25–30%. Challenges include 40% non-adherence, rural care gaps, and inconsistent protocols. Future strategies include telemedicine (45% adherence gain), genetic risk tools, and machine learning for early detection.

## 1 Introduction

Neutropenia, defined as a neutrophil count below 1,500 cells/μL in adults, is a clinically significant hematologic disorder that impairs innate immunity and elevates infection risk (1). It occurs through two main mechanisms: reduced production due to bone marrow suppression (e.g., chemotherapy, radiation, hematologic malignancies) and increased destruction caused by autoimmune processes or splenic sequestration (2–4). Infection risk increases with neutropenia severity, and patients with counts below 500 cells/μL are at especially high risk for severe bacterial and fungal infections (1, 2, 4). In oncology, chemotherapy-induced neutropenia is a major dose-limiting toxicity, leading to treatment delays and higher infection-related mortality (3, 5).

The clinical impact of neutropenia extends beyond infection to affect overall treatment outcomes and quality of life (6, 7). Febrile neutropenia (FN), defined as a single temperature >38.3 °C or sustained fever >38 °C for 1 h, is a medical emergency requiring immediate intervention (5, 7). Despite improved antimicrobial treatments, FN is still associated with 10–30% mortality (7). Outside of cancer, neutropenia linked to autoimmune diseases, chronic viral infections, or congenital syndromes presents unique diagnostic and management challenges (5). The substantial economic burden of neutropenia complications highlights the need for effective prevention and management (8).

This study examines the essential role of nursing in improving outcomes for neutropenic patients through comprehensive clinical monitoring, education, and psychosocial care. Although current guidelines focus on pharmacologic measures like granulocyte colony-stimulating factors (G-CSFs) and prophylactic antibiotics, the role of nurses in infection prevention, early detection, and patient empowerment is often underrecognized (9). Nurses are key frontline providers, responsible for implementing strict hand hygiene, environmental precautions, and infection surveillance (9, 10). Their role extends beyond inpatient care, as 60% of FN cases occur in outpatients, emphasizing the need for effective discharge planning and follow-up (10).

The complex nature of neutropenia management requires a standardized nursing framework combining evidence-based care with patient-centered education and support (11). This approach must address both physical complications and psychological distress, such as anxiety over infection risk and social isolation from protective precautions (12, 13). Structured protocols for risk assessment, education, and psychosocial care can help nurses reduce preventable complications and enhance quality of life (12, 13). This study calls for greater recognition of nursing’s role in neutropenia care and further research on care models that integrate inpatient and outpatient management.

## 2 Nursing care for patients with neutropenia

Managing neutropenic patients requires a comprehensive, evidence-based nursing strategy that focuses on four key domains: infection prevention, clinical monitoring, pharmacologic management, and nutritional support. Each component must be carefully implemented to reduce infection risk and improve outcomes in this high-risk population.

### 2.1 Infection prevention and control

Infection prevention is the cornerstone of neutropenic care, demanding strict compliance with evidence-based guidelines. The Centers for Disease Control and Prevention advises that healthcare workers perform hand hygiene using 60–95% alcohol-based hand rubs or 2–4% chlorhexidine soap, scrubbing for 15–30 s before and after patient contact (14, 15). Protective measures should include positive-pressure rooms with at least 12 air exchanges per hour and high efficiency particulate air filtration capable of removing >99.97% of particles ≥0.3 μm (14, 15). Weekly environmental surveillance cultures are recommended in high-risk units to detect potential fungal contamination (16). Early infection detection is essential, with close monitoring of temperature patterns (≥38.3 °C once or ≥38.0 °C for ≥1 h) and subtle signs such as perianal pain or gingival redness, which may precede clinical sepsis in immunocompromised patients (17).

While interventions such as high-efficiency particulate air filtration and frequent laboratory monitoring are effective, their high cost and infrastructure requirements often limit their implementation in low- and middle-income countries. In these settings, cost-effective strategies—such as natural ventilation, ultraviolet germicidal irradiation, and targeted risk-based laboratory testing—have been shown to achieve similar reductions in infection rates at lower cost (18). These approaches should be integrated with essential measures, including strict hand hygiene and environmental cleaning, to optimize infection control.

In addition to environmental cleaning and hand hygiene protocols, the routine use of medical-grade face masks is essential for neutropenic patients, caregivers, and visitors, especially during hospital visits or in poorly ventilated areas (13). Patients should wear American Society for Testing and Materials level 2 or higher surgical masks when outside protective isolation areas, while caregivers should use well-fitted surgical or N95 masks during direct contact procedures such as dressing changes or catheter care. Personal hygiene protocols recommend twice-daily bathing with antiseptic soap, daily perineal care, and avoiding shared items such as towels, razors, and toothbrushes. A structured infection control training program for caregivers should include instruction on hand hygiene techniques, correct use of personal protective equipment, symptom monitoring, and compliance with visitation restrictions during febrile episodes. Clear home-care guidance should also be provided, covering surface disinfection, food safety practices, and respiratory hygiene to minimize infection risks in immunocompromised patients.

### 2.2 Clinical monitoring and assessment

Effective clinical monitoring requires a structured approach combining laboratory tests and physical assessments. High-risk patients should undergo complete blood counts with manual differentials at least twice per week, with immediate physician notification if absolute neutrophil counts (ANC) falls below 500 cells/mm^3^ (17). The Multinational Association for Supportive Care in Cancer risk index should be routinely calculated for all FN cases to inform management decisions (19). Skin assessments should include detailed documentation of invasive device sites, with attention to intertriginous areas and pressure points. Oral assessments should use the World Health Organization Oral Toxicity Scale, with grade ≥2 mucositis (e.g., painful erythema, ulcers, difficulty eating) requiring specialized oral care interventions (20). Hemodynamic monitoring should include orthostatic vital sign assessment in febrile patients to detect early signs of septic shock.

### 2.3 Medication management

Pharmacologic management requires accurate dosing and ongoing monitoring to ensure efficacy. G-CSFs should be given subcutaneously at 5 μg/kg/day beginning 24–72 h after chemotherapy, continuing until ANC exceeds 2,000 cells/mm^3^; pediatric dosing should be adjusted to 10 μg/kg/day (3). Fluoroquinolone prophylaxis (e.g., levofloxacin 500 mg daily) should be started when ANC is expected to be <100 cells/mm^3^ for more than 7 days, with antifungal agents (e.g., posaconazole 200 mg tid or voriconazole 200 mg bid) for high-risk patients (21, 22). Administration records must accurately log dosing times for time-sensitive antibiotics (e.g., piperacillin–tazobactam every 6 h), and include therapeutic drug monitoring for agents like vancomycin (target trough 10–15 μg/mL) and aminoglycosides (23). Nurses must cross-check all medication orders with the patient’s current renal and liver function to ensure safe dosing.

### 2.4 Nutritional support

Nutritional management should balance infection control with sufficient caloric and protein intake. A neutropenic diet must exclude raw vegetables, unpeeled fruits, undercooked meats (internal temperature <165 °F), and unpasteurized dairy products (24). Protein intake should be maintained at 1.5–2.0 g/kg of ideal body weight, and branched-chain amino acid supplements may benefit patients with chemotherapy-related anorexia (25). Daily calorie intake and weekly weight measurements (with bed scales if needed) should be recorded to monitor nutritional status, and serum prealbumin levels (normal: 15–35 mg/dL) should be checked biweekly as a sensitive indicator of protein reserves (26). Patients with grade ≥2 mucositis may require temporary enteral nutrition via nasogastric tube using 1.0–1.5 kcal/mL polymeric formulas to meet at least 75% of their energy needs (27). All interventions should be coordinated with registered dietitians and guided by validated tools such as the Patient-Generated Subjective Global Assessment (28).

Adequate hydration is essential for neutropenic patients, as it supports renal clearance of chemotherapeutic metabolites and maintains mucosal hydration (24, 27). A daily intake of 30–35 mL/kg body weight is generally recommended, unless limited by conditions such as heart failure or renal impairment (27). This typically corresponds to 2.0–2.5 L per day for most adults. Patients with mucositis or persistent fever may require higher volumes, with adjustments based on serum electrolytes, urine output, and body weight changes. Nurses should monitor daily fluid balance and educate patients to recognize signs of dehydration, such as dry mucous membranes, dark urine, and orthostatic hypotension.

Evidence supports the feasibility and cost-effectiveness of home-based care protocols for pediatric patients with chemotherapy-induced FN. A prospective study conducted at a pediatric oncology center investigated the effectiveness of home-based care protocols for children with chemotherapy-induced FN. The study included six patients (aged 2–17 years) experiencing 16 FN episodes (29). Following hospital discharge, patients received prophylactic filgrastim and structured nursing interventions, which comprised daily temperature monitoring, strict aseptic care, and regular teleconsultations. All FN episodes were successfully resolved within 12 days, and no hospital readmissions occurred. Notably, the home-based care model resulted in significant cost savings, with a total expenditure of approximately $22,400, compared to $112,924 for standard inpatient management—representing an overall cost reduction of nearly 80% (29).

This comprehensive nursing protocol, based on clinical guidelines and evidence, offers a structured approach to managing neutropenic patients across all care settings. Its implementation depends on interdisciplinary coordination and continuous staff education to ensure adherence to these standards.

Special considerations are required for vulnerable populations, including pediatric, geriatric, and culturally diverse patients. For pediatric patients, age-appropriate infection prevention education, family-centered care, and nutritional interventions aligned with growth requirements are critical components of effective nursing practice (30). Geriatric patients often benefit from individualized medication dose adjustments, thorough assessment of comorbidities, and fall-prevention strategies to address frailty and polypharmacy (31). For culturally diverse patients, adopting culturally sensitive communication approaches—such as providing multilingual educational materials and integrating culturally relevant health practices—can significantly improve adherence, trust, and overall treatment outcomes (32).

Growing evidence underscores the measurable benefits of nursing interventions, showing significant improvements in both clinical outcomes and patient-centered indicators. The ESMO Clinical Practice Guidelines indicate that nurse-led infection prevention and management strategies effectively reduce the incidence and severity of FN, thereby shortening hospital stays and improving treatment continuity (33). Additionally, structured nurse-led transitional care programs have shown notable benefits, such as reducing unplanned hospital readmissions and improving patient satisfaction through coordinated post-discharge support (34). Together, these findings highlight the pivotal role of nursing care in optimizing clinical effectiveness and enhancing the overall quality of care for patients with neutropenia.

## 3 Patient and caregiver education

Patient and caregiver education is a key component of neutropenia management, significantly improving clinical outcomes by enhancing self-monitoring and promoting risk-reducing behaviors. Educational programs should target three core areas: early symptom recognition, environmental risk reduction, and safe medication management, all supported by evidence-based guidelines and validated teaching methods.

### 3.1 Self-management strategies

Structured education should provide patients with systematic infection monitoring skills, using validated tools and clearly defined thresholds for clinical action (35). Patients should be trained to use digital thermometers accurately and instructed to report any single reading ≥38.3 °C or a persistent temperature ≥38.0 °C for over 1 h to their healthcare provider (35, 36). Education should highlight subtle symptoms, such as feeling feverish without a documented fever, unexplained fatigue, or new pain at vascular access sites (36). Oral hygiene education should include step-by-step instructions on using ultra-soft toothbrushes and antimicrobial rinses, with emphasis on avoiding mucosal injury, especially in patients at risk of mucositis (37). Patients should also be taught daily skin inspection routines for high-risk areas, using visual aids to identify warning signs like erythema >2 cm or purulent drainage from wounds or catheter sites (38).

Emerging technologies can further enhance self-management strategies. An exploratory pilot study evaluated a wearable patch sensor capable of continuous body temperature and heart rate monitoring in patients with hematologic malignancies undergoing chemotherapy (39). The system demonstrated a sensitivity of 88% and a specificity of 97% in detecting FN episodes, with strong reproducibility (intraclass correlation coefficient = 0.865) (39). Notably, tachycardia was identified as a key predictor of mortality risk, underscoring the potential of integrating real-time wearable monitoring to facilitate early intervention and reduce complications.

In parallel with patient-focused strategies, caregivers play an essential role in infection prevention and treatment adherence (40, 41). They should receive explicit guidance to avoid contact with individuals exhibiting symptoms of illness and to refrain from bringing patients into crowded environments or waiting areas (40). During patient care activities, strict adherence to hygiene protocols—including handwashing before and after contact, wearing medical-grade masks, and using gloves when handling bodily fluids or changing dressings—is critical. Caregivers must also be trained to recognize early warning signs of infection, such as fever, chills, mucosal ulcers, and changes in mental status, and to report them promptly (41). The use of structured daily checklists or symptom-reporting tools can facilitate timely communication with the clinical team. Additionally, caregivers should maintain ongoing contact with healthcare providers to clarify treatment plans, discuss medication changes, and receive continuous education, especially during care transitions from hospital to home.

### 3.2 Lifestyle modifications

Risk reduction education should provide clear, actionable guidance grounded in current epidemiological data and tailored to each patient’s risk profile. Patients should be instructed to avoid high-risk indoor environments, defined as spaces with more than 10 individuals per 100 square feet or poor ventilation. Contact precautions must include maintaining a distance of ≥2 m from symptomatic individuals and avoiding contact for at least 14 days after exposure (42). Food safety guidance should include United States Department of Agriculture—recommended cooking temperatures, correct storage conditions for perishables, and the use of food thermometers when needed (24). Patients should receive categorized lists of restricted foods with safer alternatives, highlighting commonly missed risks like raw spices and unpasteurized drinks (24). Environmental measures must detail high-efficiency particulate air filtration systems with minimum efficiency reporting value ratings of 17–20, along with clear pet interaction guidelines during periods of severe neutropenia (14).

### 3.3 Medication adherence

Improving medication adherence requires a multifaceted strategy combining patient education, technology, and systematic monitoring. Competency-based training, especially with hands-on practice and real-time feedback, improves adherence and provider satisfaction (43). For therapies such as colony-stimulating factors, training should cover correct administration and how to distinguish expected effects (e.g., mild bone pain) from serious adverse events.

Technology plays a critical role in adherence. Electronic health record-integrated systems improve medication reconciliation and support timely dosing via automated alerts (44). They should include clear escalation protocols, such as alerts for abnormal vital signs or neurological changes. mHealth tools offer additional support; smartphone-based interventions have increased adherence by 42% in cancer patients (45). Educational content should be health literacy–appropriate and delivered in multiple formats for maximum effectiveness. Documenting patient understanding in a standardized manner ensures comprehension and supports long-term adherence (46).

Recent evidence further underscores the potential of digital health interventions. A nurse-led mobile health (mHealth) program for breast cancer patients significantly improved adherence and treatment continuity by integrating automated medication reminders, real-time symptom tracking, and tele-nursing support (47). Participants reported increased engagement and satisfaction, with adherence rates exceeding 90% compared to baseline (47). These findings highlight the value of mHealth platforms in promoting medication adherence and minimizing therapy interruptions among patients with neutropenia.

## 4 Psychosocial support and quality of life

Chemotherapy-induced neutropenia substantially impairs psychological wellbeing, with 40–45% of patients reporting anxiety and 30–35% experiencing depression during treatment (48). This distress is primarily caused by treatment-related side effects and heightened infection risk, which negatively impact quality of life (49). Patients often adopt maladaptive coping behaviors, such as social withdrawal (50), and 45% of caregivers report considerable stress related to infection prevention responsibilities (51). These findings emphasize the need for comprehensive psychosocial interventions targeting both patients and caregivers.

### 4.1 Evidence-based supportive interventions

Supportive interventions are most effective when delivered through a stratified approach tailored to the severity of psychological distress. For mild symptoms, nurse-led cognitive-behavioral interventions improve psychological outcomes by 45% (52). Patients with moderate distress benefit from structured psycho-oncological programs, such as the Managing Cancer and Living Meaningfully model, which is 38% more effective than standard care (53). Telehealth-based interventions significantly improve both access and adherence (80% vs. 55% with in-person therapy) (44). Artificial intelligence-matched virtual support groups show strong sustainability, with 70% retention at 6 months, and mindfulness-based interventions significantly reduce stress (effect size = 0.65) (54, 55). Regular monitoring with validated tools such as the European Organisation for Research and Treatment of Cancer Quality of Life Questionnaire–Core 30 ensures intervention fidelity and supports timely therapeutic adjustments.

### 4.2 Implementation framework

A standardized screening protocol should be implemented using the validated National Comprehensive Cancer Network Distress Thermometer at every clinical visit (56). Scores of 4 or higher should automatically initiate a referral to psychosocial services within 72 h. This process follows a standardized five-step model: identification, triage, referral, intervention, and follow-up (57).

Multidisciplinary teams should coordinate care with clearly defined roles and responsibilities. They must integrate trauma-informed care principles to address emotional needs while upholding safety measures (58, 59). Healthcare institutions should provide adequate infrastructure, including private counseling rooms and dependable telehealth platforms, to ensure equitable access to care.

Future research should focus on two priorities: (1) identifying neural biomarkers of psychological vulnerability for early risk detection (60); and (2) developing targeted interventions for high-risk patients through collaborative research initiatives (59).

## 5 Interdisciplinary collaboration

Structured interdisciplinary collaboration yields substantial clinical benefits in neutropenia management. Clinical pathway programs reduce FN complications by 25–30% by integrating hematology, infectious disease, pharmacy, and nutrition into coordinated care (61). Hospital-based multidisciplinary models lead to 18–22% reductions in readmission rates through optimized care coordination (62). Effective implementation depends on three key elements: standardized clinical protocols, robust communication systems, and clearly defined accountability measures (61, 62).

### 5.1 Role of the healthcare team

Effective neutropenia management requires a multidisciplinary team-based approach. The team should consist of hematologist-oncologists, infectious disease experts, oncology-certified nurses, clinical pharmacists, and registered dietitians, each bringing specialized knowledge (63).

Infectious disease specialists guide antimicrobial therapy, tailoring regimens to local resistance patterns and patient-specific factors (63). Clinical pharmacists optimize medication dosing, especially for colony-stimulating factors, and screen for potential drug interactions (64, 65).

Oncology nurses coordinate care through implementation of evidence-based infection control protocols (13). Registered dietitians create individualized nutrition plans, aiming for protein intake of 1.2–1.5 g/kg/day and monitor micronutrient levels (24).

### 5.2 Transitional care and follow-up

Effective transitional care programs require three core components to ensure patient safety and care continuity (66, 67). First, standardized discharge protocols should be used to confirm that patient education and follow-up plans are complete. Second, structured inter-provider communication using the Situation-Background-Assessment-Recommendation framework ensures accurate information transfer. Third, telehealth follow-up should be scheduled within 48–72 h after discharge to assess patient condition (66).

Post-discharge monitoring should include defined laboratory thresholds, such as ANC <500 cells/μL, to prompt clinical reassessment (68). Programs integrating these elements have shown 35–40% reductions in unplanned readmissions and 25% improvement in patient satisfaction (34).

## 6 Challenges and future directions

Neutropenia management involves persistent challenges that require structured solutions, while emerging innovations offer new opportunities to improve patient outcomes. This section examines current barriers and highlights potential innovations in neutropenia care.

### 6.1 Barriers to optimal care

Patient- and system-level barriers continue to hinder effective neutropenia care. Approximately 40% of patients fail to follow neutropenic precautions, mainly due to low health literacy and financial barriers (69, 70). Poor coordination between primary and specialty providers, coupled with limited reimbursement for patient education, worsens care disparities (71). Rural patients frequently experience delays due to limited access to hematology/oncology specialists (72). Inconsistent implementation of evidence-based protocols across institutions is associated with a 25% increase in complications among vulnerable groups (73).

### 6.2 Innovations and future directions

Telemedicine integrated with mobile health (mHealth) platforms represents a key innovation in neutropenia care, with studies demonstrating a 45% improvement in patient adherence (74). These technologies enable real-time symptom tracking, automated febrile alerts, and remote consultations, thereby improving care accessibility and timely intervention. Notably, decision-support algorithms embedded within mHealth applications can guide caregivers in determining when to escalate care based on reported symptoms or abnormal vital signs. In parallel, advancements in pharmacogenomic and germline genetic testing offer the potential for personalized prophylactic strategies and accurate prediction of chemotherapy-induced neutropenia risk, allowing for earlier and more targeted interventions (75). Current research priorities focus on developing individualized nursing care bundles, optimizing antimicrobial prophylaxis duration, and implementing digital monitoring tools to facilitate early infection detection and clinical response (76, 77). From an implementation science perspective, frameworks such as Reach, Effectiveness, Adoption, Implementation, and Maintenance offer a structured model for evaluating the feasibility, adoption, and scalability of these innovations across diverse healthcare settings. Emerging directions include the use of machine learning algorithms for real-time risk prediction and the development of validated patient-reported outcome measures specific to neutropenia-related quality of life domains (76, 78).

## 7 Summary

This study establishes the critical role of nursing interventions in managing neutropenia through evidence-based practice. Structured nursing care encompassing infection surveillance, protective measures, and psychosocial support demonstrates significant clinical benefits, reducing complications by 30–40% and improving quality of life by 25–35%. Three key priorities emerge for advancing care quality: First, implementing standardized simulation training programs to ensure protocol competency. Second, developing integrated multidisciplinary care pathways. Third, promoting patient empowerment through shared decision-making tools. Future research should focus on two critical areas: validating nursing-sensitive outcome measures and evaluating digital health innovations in care delivery. Continuous competency assessment and quality improvement initiatives remain essential for sustaining these clinical gains.
